# Genetic Variation in *LRP1* Associates with Stanford Type B Aortic Dissection Risk and Clinical Outcome

**DOI:** 10.3390/jcdd9010014

**Published:** 2022-01-05

**Authors:** Philipp Erhart, Daniel Körfer, Caspar Grond-Ginsbach, Jia-Lu Qiao, Moritz S. Bischoff, Maja Hempel, Christian P. Schaaf, Armin Grau, Dittmar Böckler

**Affiliations:** 1 Department of Vascular and Endovascular Surgery, Heidelberg University Hospital, 69120 Heidelberg, Germany; Daniel.Koerfer@med.uni-heidelberg.de (D.K.); Caspar.Grond-Ginsbach@med.uni-heidelberg.de (C.G.-G.); jiaiu.qiao@stud.uni-heidelberg.de (J.-L.Q.); Moritz.Bischoff@med.uni-heidelberg.de (M.S.B.); Dittmar.Boeckler@med.uni-heidelberg.de (D.B.); 2 Institute of Human Genetics, Heidelberg University, 69120 Heidelberg, Germany; Maja.Hempel@med.uni-heidelberg.de (M.H.); Christian.Schaaf@med.uni-heidelberg.de (C.P.S.); 3 Department of Neurology, Community Hospital Klinikum der Stadt Ludwigshafen am Rhein, 67063 Ludwigshafen, Germany; GRAUA@KLILU.de

**Keywords:** aortic dissection, genetics, risk variant, *LRP1*, aorta

## Abstract

Genetic variation in *LRP1* (low-density lipoprotein receptor-related protein 1) was reported to be associated with thoracic aortic dissections and aneurysms. The aims of this study were to confirm this association in a prospective single-center patient cohort of patients with acute Stanford type B aortic dissections (STBAD) and to assess the impact of *LRP1* variation on clinical outcome. The single nucleotide variation (SNV) rs11172113 within the *LRP1* gene was genotyped in 113 STBAD patients and 768 healthy control subjects from the same population. The T-allele of rs11172113 was more common in STBAD patients as compared to the reference group (72.6% vs. 59.6%) and confirmed to be an independent risk factor for STBAD (*p* = 0.002) after sex and age adjustment in a logistic regression model analyzing diabetes, smoking and hypertension as additional risk factors. Analysis of clinical follow-up (median follow-up 2.0 years) revealed that patients with the T-allele were more likely to suffer aorta-related complications (T-allele 75.6% vs. 63.8%; *p* = 0.022). In this study sample of STBAD patients, variation in *LRP1* was an independent risk factor for STBAD and affected clinical outcome.

## 1. Introduction

Thoracic aortic dissection (TAD) is a life-threatening disease caused by cardiovascular risk factors such as arterial hypertension or smoking. Degeneration of the tunica media vascular wall layer due to atherosclerosis or connective tissue diseases predispose individuals to acute aortic syndromes. Epidemiological studies reveal a TAD incidence of 14 per 100,000 persons per year, of which 30% are supposed to be Stanford type B aortic dissections (STBAD) [1,2].

With increasing use and availability of genetic testing and genome-wide association studies (GWAS), it is estimated that hereditary factors play an important role in the etiology of thoracic aortic aneurysms and dissections (TAAD). In contrast to rare pathogenic variants that impair the connective tissue structure of the vascular system and cause inherited disorders such as Marfan, Ehlers–Danlos or Loeys–Dietz syndrome, common genetic variants also occur in the general population, such as single-nucleotide variations (SNV) or genomic copy number variations (CNV), which slightly increase the risk for TAAD [3,4].

Genetic testing might benefit the diagnosis, surveillance and treatment of patients with acute aortic syndromes. It is estimated that in more than 20% of TAAD a hereditary background with fibrillin-1 (Marfan syndrome) or other autosomal dominant disorders without syndromic features can be found in familial TAAD [5]. Ongoing research for aortic dissection identified mutations in genes involved in smooth muscle cell (SMC) contraction, extracellular matrix composition or the transforming growth factor beta (*TGF-β*) signaling pathway [6,7,8,9].

Gene panel analysis for TAAD is restricted to validated findings in predefined genes, enabling diagnostics and genetic counseling. However, genetic variations, such as CNVs or SNVs, might be enriched in patients suffering TAAD, which are not routinely diagnosed by standardized gene panel diagnostics and are even found in genetical segments not related to connective tissue coding proteins. 

In a genome-wide association study analyzing genotypes of 753 individuals with sporadic aortic dissection, genetic variation within the low-density lipoprotein receptor-related protein 1 (*LRP1*) was associated with TAAD development [4]. Experimental studies suggested that deletion of *LRP1* leads to impaired SMC contractility and might cause cardiomyopathy, ascending aortic aneurysms and aortic insufficiency [10,11,12]. Genetic variation in the *LRP1* gene was associated with acute aortic dissection [4], cervical artery dissection and migraine [13].

The T-allele of rs11172113 was more common in individuals with aortic dissections compared to a reference population from the Atherosclerosis Risk in Communities (ARIC) study [4]. According to the SNPedia platform (www.snpedia.com) as at 15 November 2021, the odds ratio for the rare rs11172113 C-allele was 0.9 (CI: 0.87–0.93, *p* = 4.3 × 10^−9^). 

The aim of this study was to (1) confirm that the rs11172113 variant within the *LRP1* gene is associated with STBAD; (2) analyze whether the association of rs11172113 with STBAD is independent from other risk factors; and (3) explore whether rs11172113 affects the clinical outcome of STBAD patients. 

## 2. Materials and Methods

### 2.1. Study Population

Patients with acute sporadic STBAD were enrolled prospectively between the years 2000 and 2021 at the Department of Vascular and Endovascular Surgery at Heidelberg University Hospital. The following inclusion criteria were used for all patients: (1) STBAD was diagnosed by a helical computer tomography angiography (CTA) using a 1 mm sliced electrocardiogram triggered aortic protocol; and (2) blood EDTA samples could be collected from all patients for genetic testing. Exclusion criteria were (1) a history of (chronic) aortic dissection; (2) other acute aortic pathologies, such as thoracic aortic aneurysms, intramural hematomas, penetrating aortic ulcerations or traumatic aortic injuries; and (3) genetically diagnosed hereditary connective tissue disorders prior to the STBAD event (e.g., Marfan syndrome). From the initial STBAD population all patients younger than 45 years received extensive genetic testing. Four patients with pathogenic findings were excluded from this study [14]. According to the Stanford classification, all patients had proximal entry tears starting at the level or distally from the left subclavian artery (Stanford type B aortic dissection, STBAD). From all patients clinical data were collected, including comorbidities and aortic specific parameters from follow-up investigations. Arterial hypertension and diabetes mellitus were registered based on medication data. Positive smoking status was defined as smoking until at least six months before hospitalization. Follow-up was not documented in 7 patients (6.2%). For these patients, 0 years of follow-up was entered as a dummy variable.

The clinical course of the aortic dissections was classified as either uncomplicated or complicated, using the following criteria:

- Uncomplicated STBAD: Succesfull conservative treatment without initial or developing criteria for surgical intervention during follow-up.
- Complicated STBAD: Initial or developing criteria needing surgical intervention by thoracic endovascular aortic repair (TEVAR) or open surgery. Criteria for surgical intervention included: therapeutic resistant arterial hypertension/increasing pleural effusion and/or persistent pain, aortic diameter progression, radiomorphologic rupture signs, dissection related malperfusion of organs/extremities or stroke.

### 2.2. Control Reference Group for LRP1 Testing

From the “genetic and socioeconomic determinants of ischemic stroke” GENESIS study [15], 768 healthy individuals were genotyped for the same *LRP1* rs11172113 SNV variant and used as a control reference group. All patients and controls were of the white Caucasian ethnicity from mid-west Germany. Variant rs11172113 was genotyped by restricted length fragment polymorphism analysis using standard laboratory protocols [14].

All patients gave their written consent for genetic testing and study participation. The study was approved by the local Medical Ethics Committee of the University of Heidelberg, Germany (reference numbers: S-301/2013 and amendment September 2020; S-190/2004; S-551/2018).

### 2.3. Statistics

For the univariate analysis, a *t*-test (age), Chi-square test (sex, hypertension, diabetes mellitus, current smoking and genotypes) and Mann–Whitney test (follow-up time) were used. To test the independence of the association between *LRP1* and STBAD, a logistic regression model was developed with diseases state (case versus control) as dependent variable and the *LRP1* number of T-alleles and risk factors (hypertension, diabetes mellitus and current smoking), incorporating age and sex, as determinants. A second logistic regression model was build to analyze the association between *LRP1* genotype and complications. In this model, the occurrence of complications was the dependent variable and age, sex, length of follow-up and *LRP1* genotype were entered as predictors. Since the odd ratios of the number of T-alleles are difficult to interpret, logistic regression models with a binary *LRP1* variable (TT-allele versus CT + CC-allele) were performed. 

## 3. Results

Patients’ characteristics, comorbidities and *LRP1* genotypes are displayed in Table 1. Both arterial hypertension and the T- allele of rs11172113 were significant and independent risk factors for STBAD. The T-allele rs11172113 was more common in the STBAD group (72.6% vs. 59.6%, *p* < 0.003) as compared to the control group. Logistic regression analysis confirmed the T-allele as an independent risk factor for STBAD. 

From 113 STBAD patients clinical and radiomorphological data were collected at disease onset and during follow-up investigations. A total of 7 (6.2%) patients were lost for follow-up investigations. A total of 27 patients received CTA controls within one year and 79 patients received CTA controls within a follow-up period beyond one year (mean 3.9 (0–22) years).

The clinical course of patients was categorized as uncomplicated in 29 patients and complicated in 84 patients. In total, 3 patients of the uncomplicated and 4 patients of the complicated STBAD group were lost for follow-up investigations. Significantly more aorta-related complications were detected in patients with the TT genotype (*p* = 0.021). Within the STBAD group, 11 aorta-related deaths were registered during follow-up analysis, 7 of whom had the TT genotype.

Additionally, poorer clinical outcome with more aorta-related complications was observed in young (*p*= 0.038) and female patients (*p=* 0.028) (see Table 2).

Age at STBAD disease onset, however, was not independently associated to the rs11172113 *LRP1* genotype (CC and CT genotype versus TT genotype: mean 56.8 ± 12.1 years/56.0 ± 11.0 years; *p* = 0.759).

## 4. Discussion

This study confirms that the T allele of SNV rs11172113 is an independent risk factor for sporadic Stanford type B aortic dissection. In accordance to the observations by Guo et al. [4], the minor C allele protects individuals against STBAD. 

Our data moreover indicate that this SNV affected the clinical outcome of STBAD patients, due to a higher rate of aorta-related complications. Poorer clinical outcome was observed in young patients, females and patients with the T-allele.

It has previously been reported within the Swedish National Patient Register and Cause of Death Register that the 30-day mortality after surgical repair for both Stanford type A and B aortic dissections was higher in women as compared to male patients [16]. Age of STBAD disease onset might reflect the pathophysiological distinction of genetic connective tissue disorders in younger from atherosclerosis in elderly patients. 

Variant rs11172113 is located within an intronic region of the *LRP1* gene. Genetic variants within introns can affect the regulation of gene expression, can have effects on splicing, but in most cases, the functional consequences of intronic variants are still unknown [17].

In contrast to pathogenic coding variants leading to connective tissue diseases, such as Marfan syndrome (*FBN1* gene), Ehlers–Danlos syndrome (collagen-encoding genes) or Loeys–Dietz syndrome (*TGF-β* receptor-encoding genes) [3], genetic single-nucleotide variations in the interaction with environmental risk factors or other genetic modifiers may increase the risk for STBAD and affect the clinical outcome of patients. Genetic variants for STBAD mainly affect the genes for connective tissue integrity, vascular smooth muscle cell function and TGF beta signaling [6,7,8,9]. 

Chang et al. investigated in 159 patients the association of *MYH11* and *TGF-β* genetic variations with STBAD and clinical outcome of patients. Genetic variants in *MYH11* (rs115364997) and *TGFBR1* (rs1626340) were identified as independent genetic risk factors for STBAD. The *MYH11-*(rs115364997) and *TGFB1* (rs1800469) genetic variants were prognostic parameters for higher mortality and recurrent chest pain in STBAD patients [18]. Mid- and long-term data for genotyped STBAD patients are lacking to confirm the prognostic genetic markers that may influence the clinical course of STBAD disease.

Based on the genetic variants in the *LRP1* gene, either protective or threatening effects were observed for vascular diseases so far [4,19]. In the same *LRP1* gene, another SNV rs1466535 located in an adjacent haplotype block to rs11172113 was associated with an increased risk of abdominal aortic aneurysm formation [4,20]. 

In an experimental animal model, knock out of the *LRP1* gene resulted in the development of tortuous aortas and elevated TGF-β signaling [21]. The described susceptible locus in intron 1 of the *LRP1* gene was previously described by two independent genome-wide association studies to cause abdominal aortic aneurysm formation, hence highlighting the importance of *LRP1* for vascular smooth muscle cell contraction and prevention of aneurysmal disease [10,20,22]. Repressing *LRP1* translational expression results in excess accumulation of matrix metalloproteinase (MMP) 9 in abdominal aortic vascular tissue, degrading the extracellular matrix components and consequently promoting aneurysm formation [23]. These observations indicate that *LRP1* dysfunction might provoke aortic disease development irrespective of the known candidate genes. 

Additional SNVs associated with TAAD are rs10757278 on chromosome 9p21 [24] or rs514921 in the MMP1 gene [25]. In the latter SNV, the minor allele was associated with a more favorable clinical outcome of TAAD patients. 

CNVs such as the duplication on the chromosome 16p13.1 containing the *MYH11* gene are associated with TAAD and neuropsychiatric disorders [26,27]. Additionally, genetic variants in noncoding micro-RNAs (miRNAs) controlling gene expression, may cause TAAD development via mitogen-activated protein kinase (MAPK) signaling or via specific MMPs [28,29]. 

From family aggregation studies, it has been estimated that 20% of TAAD are hereditary and due to syndromic single-gene disorders (e.g., Marfan syndrome) or without syndromic features usually following an autosomal-dominant pattern of inheritance [5]. Gene variations related to TAAD are routinely diagnosed with gene panels including, i.a., *TGFBR1*, *TGFBR2*, *SMAD3*, *MYH11*, *ACTA2*, *FBN1* and *MYLK* genes [19]. However, further genetic findings, including genes beyond the aforementioned candidate genes, as well as SNVs, CNVs or micro-RNA alterations, are often unnoticed in clinical practice, rather being of scientific interest at present as further validation is ongoing. Whole genome sequencing analysis or gene panel analysis through next-generation sequencing [30] may offer a more detailed investigation of the genetic risk variants for STBAD. In addition to the diagnostics of the known pathogenic STBAD candidate gene variants enabling genetic counseling and predictive testing of family members, genetic risk variants may play an important role in a more patient-specific risk stratification for STBAD in the future.

The small STBAD cohort size is a limitation of this study. GWAS for TAAD usually lack clinical follow-up data to evaluate the effect of genetic variants on disease progression and clinical outcome of patients. Genome-wide analysis and validation of genetic variants for TAAD contribute to epidemiologic and pathophysiologic insights. The *LRP1* variant was selected for our study as it was associated with a broad spectrum of vascular pathologies, including cervical artery dissection [31], fibromuscular dysplasia [32], migraine [33] and aortic aneurysms [34]. 

It is a strength of this study that Stanford type B aortic dissections were investigated as specific disease entity in contrast to previous studies merging thoracic aortic aneurysms and dissections (TAAD), intramural hematomas, penetrating aortic ulcers or even Stanford type A aortic dissections. In contrast to the most GWAS for TAAD, a detailed documentation of follow-up investigations enabled for the first time the correlation of the *LRP1* s11172113 SNV variation to the clinical outcome of STBAD patients.

The survival rate of STBAD is estimated to be 89.4% at 5 years and 71.8% at 10 years [35]. Although we observed a higher proportion of deaths in STBAD patients with the rs11172113 TT genotype, larger study samples with clinical follow-up data are needed to correlate genotypic findings to clinical outcome parameters. The combination and interpretation of multiple genetic risk variants (polygenic score) might be a prognostic marker for clinical outcome and aorta-related mortality in STBAD patients. Future genotyping studies of patients with aortic disease should differentiate among the heterogenous aortic pathologies, such as aneurysms, Stanford type A and B aortic dissections, penetrating ulcers and intramural hematomas, considering atherosclerosis and connective tissue disorders as different etiologies, and ensure high quantity/quality follow-up data to investigate the effect on clinical disease progression.

Genetic diagnostics and counseling should be offered to STBAD patients with disease onset under 45 years and suspected disease heredity in accordance to the recommendations by the European Society for Vascular Surgery [14,36]. Genome-wide analysis should be considered in selected TAAD when targeted genetic testing does not provide a genetic diagnosis, potentially leading to better understanding, counseling, and an improvement of patient-centered precision medicine. 

## 5. Conclusions

This study confirms previous observations that the *LRP1* rs11172113 SNV is an independent risk factor not only for TAAD but specifically for STBAD. Our study furthermore suggests that genetic variation in *LRP1* might affect the clinical outcome and is associated with more aorta-related complications. These results encourage further genetical evaluation of aortic pathologies for clinical risk assessment. 

## Figures and Tables

**Table 1:** Univariate analysis and multivariate logistic regression analysis adjusted for sex and age. Age and age at disease onset in years. Other parameters are given in total amount and percentage values. STBAD: Stanford type B aortic dissection; *LRP1*: low-density lipoprotein receptor-related protein 1; SD: standard deviation.

	Univariate Analysis			Multivariate Analysis	
	Control Group	STBAD Group	*p*-Value	*p*-Value	Odds Ratio
Total number	768	113			
Female sex	445 (57.9%)	35 (31.0%)			
Age (mean ± SD)	67.4 ± 10.1	56.4 ± 11.5 *			
Arterial Hypertension	508 (66.1%)	101 (89.4%)	<0.001	<0.001	15.4 (7.2–32.6)
Diabetes mellitus	132 (17.2%)	22 (19.5%)	0.053	0.429	1.3 (0.7–2.4)
Smoking history	109 (14.2%)	31 (27.4%)	0.497	0.454	1.2 (0.7–2.2)
*LRP1* CC genotype	114 (14.8%)	8 (7.1%)	<0.001 ** 0.003 ***	0.002 ** 0.004 ***	1.8 (1.3–2.6) ** 2.0 (1.3–3.2) ***
*LRP1* TC genotype	393 (51.2%)	46 (40.7%)			
*LRP1* TT genotype	261 (33.9%)	59 (52.2%)			

* For the STBAD group the age at disease onset is given. ** Number of T-alleles (0,1 or 2). *** CC and CT genotypes versus TT genotype.

**Table 2:** Comparison of the STBAD LRP1 genotypes with clinical parameters and disease outcome.

	Univariate Analysis			Multivariate Analysis	
	Uncomplicated STBAD	Complicated STBAD	*p*-Value	*p*-Value	Odds Ratio
Total number	29	84			
Follow-up in years (median, IQR)	2.0 (5)	2.0 (6)	0.669	0.529	1.0 (0.9–1.2)
Age at disease onset (mean, ± SD)	60.3 (±12.8)	55.0 (±10.8)	0.037	0.038	0.96 (0.92–1.0)
Female sex	5 (17.2%)	30 (35.7%)	0.070	0.028	3.8 (1.2–12.6)
*LRP1* CC genotype	3 (10.3%)	5 (6.0%)	0.084 * 0.077 **	0.022 * 0.021 **	2.5 (1.1–5.3) * 3.3 (1.2–9.1) **
*LRP1* TC genotype	15 (51.7%)	31 (36.9%)			
*LRP1* TT genotype	11 (37.9%)	48 (57.1%)			

* Number of T-alleles (0,1 or 2). ** CC and CT genotypes versus TT genotype. STBAD: Stanford type B aortic dissection; *LRP1*: low-density lipoprotein receptor-related protein; IQR: interquartile range; SD: standard deviation.

## Data Availability

The data are available in the manuscript and on personal request to the corresponding author.
